# Strain-Engineered Tetragonal Phase and Ferroelectricity in GdMnO_3_ Thin Films Grown on SrTiO_3_ (001)

**DOI:** 10.1038/s41598-019-55227-2

**Published:** 2019-12-10

**Authors:** P. Machado, F. G. Figueiras, R. Vilarinho, J. R. A. Fernandes, P. B. Tavares, M. Rosário Soares, S. Cardoso, J. P. B. Silva, A. Almeida, J. Agostinho Moreira

**Affiliations:** 1 IFIMUP and Departamento de Física e Astronomia, Faculdade de Ciências, Universidade do Porto. R. Campo Alegre, 687, 4169-007 Porto, Portugal; 2 CQVR & Physics Department, University of Trás-os-Montes e Alto-Douro, Ap. 1013, 5001-801 Vila Real, Portugal; 3 CQVR & Chemistry Department, University of Trás-os-Montes e Alto-Douro, Ap. 1013, 5001-801 Vila Real, Portugal; 4 CICECO & LCA, University of Aveiro, 3810-193 Aveiro, Portugal; 5 INESC-MN e Instituto Superior Técnico, Universidade de Lisboa, Rua Alves Redol 9, 1000-029 Lisboa, Portugal; 6 Centro de Física das Universidades do Minho e do Porto (CF-UM-UP), Campus de Gualtar, 4710-057 Braga, Portugal

**Keywords:** Materials science, Physics

## Abstract

A previously unreported tetragonal phase has been discovered in a epitaxially strained GdMnO_3_ thin films deposited on (001)-oriented SrTiO_3_ substrates by radio frequency (RF) magnetron sputtering. The tetragonal axis of the films grown up to a 35 nm thickness is perpendicular to the film surface and the basal lattice parameters are imposed by the cubic structure of the substrate. Furthermore, the emergence of a spontaneous electric polarization below ~32 K points to the stabilization of an improper ferroelectric phase at low temperatures, which is not observed in bulk GdMnO_3_. This work shows how strain engineering can be used to tailor the structure and properties of strongly correlated oxides.

## Introduction

Strain engineering has been an interesting way to control the physical properties of materials at the nanoscale level, such as thin films that can withstand tensile or shear stresses up to a significant fraction of their ideal strength^1–3^. The structural distortions induced by strain may stabilize new crystallographic structures and thermodynamic phases, some of them not found in the bulk material, promoting new functionalities of materials^2–5^. To attain significant effects of strain on the physical properties, in addition to a good connectivity between film and substrate, a strong coupling between lattice and other degrees of freedom, like electronic charge, spin and orbital, is required. In this regard, rare-earth manganites (*R*MnO_3_, *R* a trivalent rare-earth cation) are attractive candidates for strain-engineered designed properties as they fulfill this requirement.

Rare-earth manganites exhibit a large set of physical properties, including colossal magnetoresistance, charge-orbital ordering, different magnetic structures and multiferroicity^6^. Because the magnetism and transport properties of these compounds scale with the Mn-O-Mn bond angle, which defines the balance between competitive electronic and magnetic interactions, these materials are highly sensitive to external parameters which can distort the crystallographic structure, like pressure, applied magnetic/electric fields and epitaxial strain^6^. GdMnO_3_ (hereafter designated by GMO) has attracted much attention with the aim to strain-engineering its properties, as its Mn-O-Mn bond angle sets it at the border line between spontaneous multiferroics TbMnO_3_ and DyMnO_3_, and the magnetic-field induced multiferroic EuMnO_3_^7–9^.

At room conditions, bulk GMO is orthorhombic with P*nma* symmetry^9,10^. The magnetic phase sequence of GMO can be summarized as follows^7–9^. At T_N_ = 42 K, the Mn^3+^ spins order in a collinear incommensurate antiferromagnetic structure, with modulation wave vector along the *a*-axis. At T’ =23 K there is a transition into a canted A-type antiferromagnetic structure. The Gd^3+^ spins only order below T_N_^Gd^ ~ 5.1 K. Concerning the existence of a spontaneous ferroelectric phase  in bulk GMO, contradictory results have been reported. Following Kimura *et al*., GMO exhibits a spontaneous ferroelectric polarization along the *c*-axis, between 5.1 K and 8.2 K, even in the absence of a magnetic field^9^. When an applied magnetic field is applied along the *a*-axis, the temperature range of stability of the ferroelectric phase increases up to 15 K, stabilizing a commensurate magnetic structure similar to the one found in TbMnO_3_ and DyMnO_3_^9^. On the other hand, Noda *et al*. reported a finite polarization below 13 K in the absence of an external magnetic field^10^.

GMO thin films have been deposited onto different substrates by using both chemical and physical routes^11–19^. Up to now, two different crystal symmetries were reported for GMO thin films. Orthorhombic GMO thin films have been the most frequently obtained^14–19^. GMO thin films, with 10 nm thickness, were successfully deposited on oriented (010)-YAlO_3_ substrates by pulse laser deposition method^15,16^. The films are orthorhombic at room conditions and exhibit a polar phase below 30 K, with a remanent polarization about ~1μC/cm^2^ at 10 K. The rather large value of the electric polarization has been ascribed to the stabilization of an antiferromagnetic phase, characterized by an incommensurate Mn^3+^ spin ordering, which successively locks into a commensurate E-type state^16^. GMO films onto (100)-oriented Si substrate, prepared by sol-gel method, with 230 nm average thickness, are paramagnetic above 80 K, showing a ferromagnetic response below 15 K. The temperature dependence of the magnetization, measured in both zero-field cooling (ZFC) and field-cooling (FC) conditions, shows an anomaly at T~27 K that has been assigned to the magnetic transition taking place in this temperature range in the bulk material^18,19^. More recently, a ferroelectric state, with rather high Curie temperature (T_C_ = 75 K) and a strong ferromagnetic component emerging below 105 K, was reported in orthorhombic GMO thin films (average thickness 110 nm) deposited by pulsed laser method onto (001)-oriented SrTiO_3_ substrates^17^. A stabilization of an *ac*-plane spiral spin order below 105 K was proposed to explain the stabilization of the ferroelectric phase and a nano-scale twin-like domain structure was appointed as essential for the rather high temperature ferroelectric and ferromagnetic phases observed in the orthorhombic GMO films^17^.

Hexagonal GMO thin films have also been reported in literature^12,13^. GMO thin films deposited onto YSZ(111) substrates, with average thickness 67 nm, exhibit enhanced ferromagnetic properties^13^. The temperature dependence of the magnetization measured in ZFC conditions shows clear anomalies at 56 and 40 K, respectively, while the FC curve exhibits a broad peak at about 30 K. The anomaly at 56 K was assigned to the antiferromagnetic ordering of the Mn^3+^ spins in the in-plane triangular lattice, and the anomaly at 40 K was ascribed to the Mn^3+^ spin-reorientation transition^13^.

The aforementioned results were obtained using GMO films with thicknesses larger than 60 nm, where relaxed structures are expected to occur. In this work, we report an earlier not reported tetragonal phase in epitaxially strained GdMnO_3_ films onto (001)-oriented SrTiO_3_ substrate, with a thickness up to 35 nm, deposited by magnetron RF-magnetron sputtering technique. The polar properties are studied by measuring the pyroelectric current using different experimental conditions.

The presented study is just focused on 35 nm thick GMO thin films. This specific choice is based on preliminary XRD data (not shown here) obtained in films with thicknesses from 20 nm up to 150 nm. While for thicknesses up to 35 nm the films are tetragonal, above 35 nm they exhibit an orthorhombic symmetry generally found in both GMO bulk and relaxed thin films. As the latter case does not bring any novelty, we did not include the study of relaxed thin films in this work. From the tetragonal films, we choose the thickest film in order to ensure the required experimental conditions to characterize their macroscopic properties. The obtained results are discussed involving a comparison with previous results in orthorhombic GMO films deposited onto similar substrate.

Details of sample morphology and chemical composition analysis are available in *Supplementary Material*.

**Figure 1:**
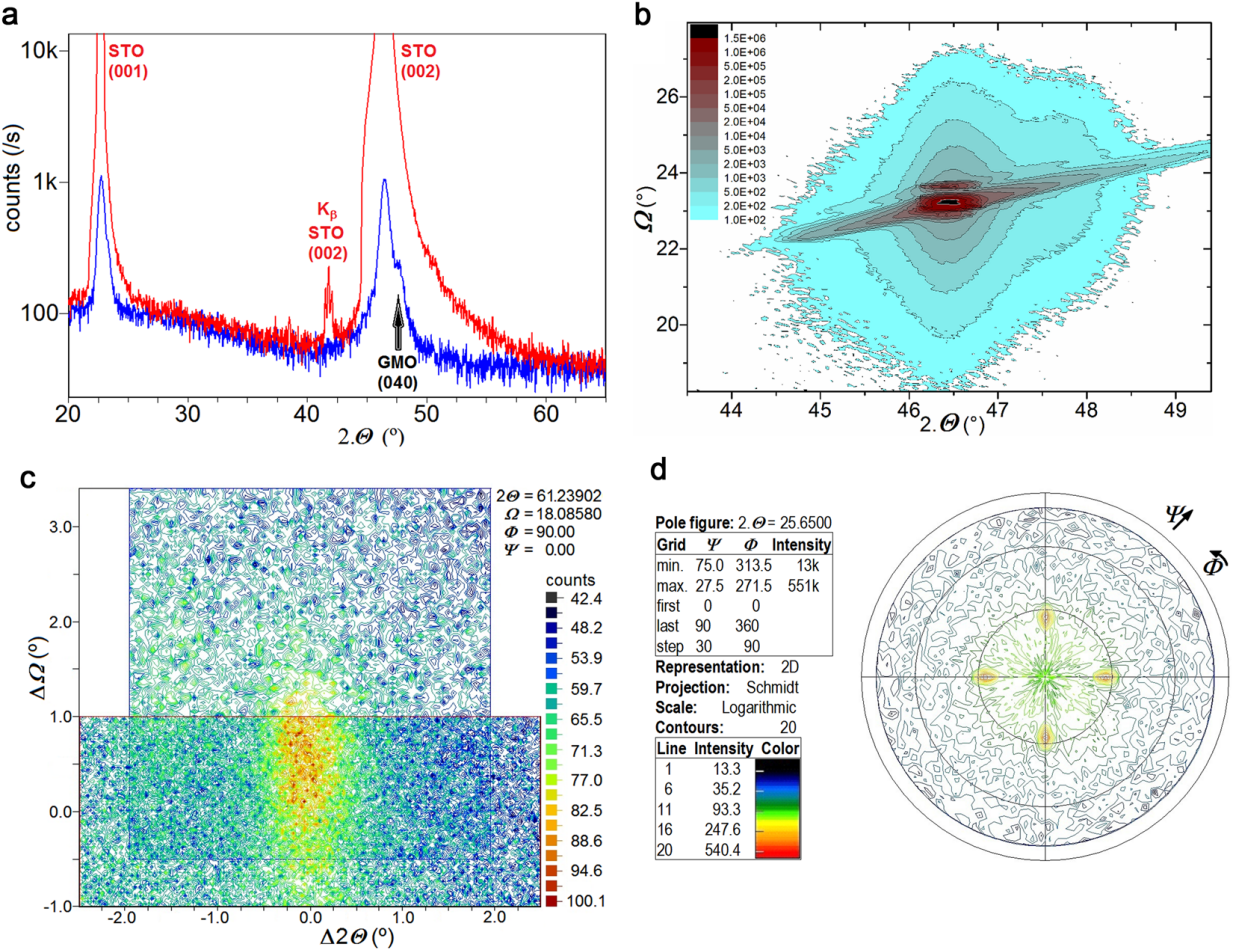
(**a**) Detail of the conventional (gonio) XRD pattern of sample “GMO/STO(001)”, red) standard and blue) with 1.5° angular off-set for maximizing thin film reflections. Projection in the (***Θ***, ***Ω***) space coordinates of the symmetric reciprocal space map centered at (**b**) 2***Θ*** = 46.47° and **Ω** = 23.23° and (**c**) at 2***Θ*** = 61.24° and ***Ω*** = 18.09°. (**d**) Symmetric pole figure obtained at 2Θ = 25.65° corresponding to the 4 reflections of GMO (111) planes.

**Figure 2:**
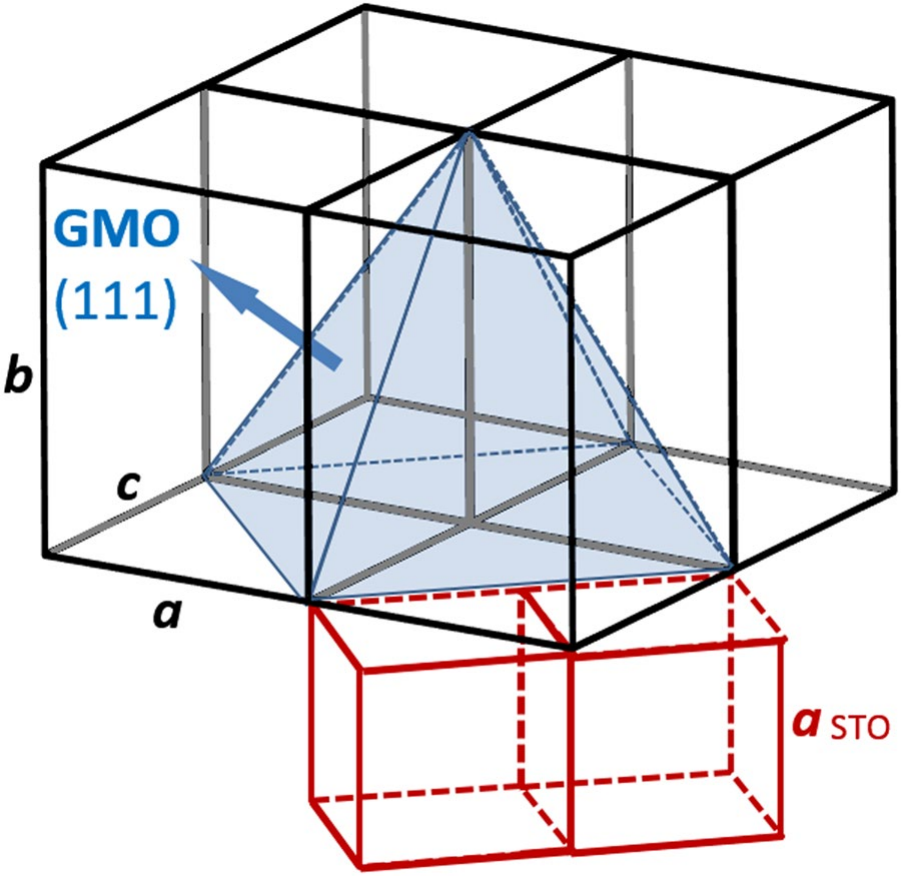
Schematics of the relative orientation of the tetragonal GMO thin film onto STO substrate and the 4 reflections from planes (111).

Figure 1(a) shows a representative XRD pattern in the 20–65° 2Θ-range, recorded at room conditions, of the 35 nm GMO thin film deposited onto (001)-oriented SrTiO_3_ (hereafter designated by STO(001)). The XRD pattern exhibits the strongest Bragg peaks approximately centered at 2Θ = 22.53° (001), 46.38° (002), 72.55° (003) and 104.24° (004), which are indexed to SrTiO_3_ crystal, confirming the high quality and the (001)-orientation of the substrate, with *a*_STO_ = 3.905(0) Å^17,20^. One significant diffraction peak centered at 2Θ = 47.60° spreading ± 0.03° (*d* = 1.91 ± 0.01 Å), is indexed to reflections from planes (040) of the GMO film, ascribing *b* = 7.64(0) Å^21^. It is worth to stress that the XRD pattern recorded for this films does not clearly shows the Laue´s oscillations. The lack of interference fringes can result from the high quality of the epitaxial growth of the GMO film, due to no relevant interface mismatch with the STO(001) substrate^22^. Figure 1(b) shows the symmetric reciprocal space map centered at the main reflection peak arising from (002) STO planes, located at 2Θ = 46.47° and Ω = 23.23°. The single satellite reflection derived from the (040) GMO planes is only + 0.4° offset in Ω and exhibits a small dispersion ∆(2Θ) ≤ 0.6°, closely conforming the profile of the substrate peak, which is indicative of the tight epitaxial fitting of the film to the substrate. Figure 1(c) shows the asymmetric map centered at 2Θ = 61.24° and Ω = 18.09°, depicting a single peak, with a limited spread with ∆(2Θ) and ∆Ω ≤ 1°, indexed to the (203) planes of the GMO film. All the peaks arising from the GMO film are well defined, relatively narrow and do not show mirroring, broadening nor diverging features from the respective centers. Consequently, there are no typical indications of maculae, neither domains due to a twinned crystalline structure, not even of significant lattice relaxation throughout the whole film thickness. Therefore, the film grows epitaxially in a single uniform crystallographic phase. The symmetric pole figure presented in Fig. 1(d), centered at 2Θ = 25.65° (*d* = 3.470 Å), encompasses and maximizes the reflections from the (111) planes of the GMO film, whereas the STO (111) reflections are found at 2Θ = 39.96°. The projected 4-fold peaks clearly configure a square, which evidences that the in-plane *a* and *c* parameters of the GMO film are forcibly identical. Moreover, these well-defined peaks are precisely aligned with the substrate edges corresponding to the respective (100)_STO_ and (010)_STO_ axes, which is a clear indication that the in-plane *a* and *c* axes of GMO film are oriented at 45° to the in-plane STO axes. Accordingly, *a* and ***c*** are estimated to be 5.48(9) Å, approximately (−0.6%) the diagonal spacing √2.*a*_STO_ = 5.522(5) Å of the substrate. These sets of results are unequivocally consistent with a tetragonal symmetry with preferential oriented growth along *b* axis perpendicular to the substrate surface, as schematically represented in Fig. 2. The present study demonstrates that the ~35 nm GMO thin films obtained in this work do not follow the expected orthorhombic symmetry found in GMO thin film with larger thickness, obtained using pulse laser deposition technique^17^. Possible effects of oxygen off-stoichiometry, that could be traced to an excess expansion of *b* parameter^23^, are negligible and within the same ~1% error margin of the calculated cell parameters. Bragg peaks that are ascribed to other crystallographic and chemical phases could not be detected.

The epitaxial GMO thin film has an overall unit cell volume constrain of ~0.7%, in comparison to the respective cell parameters of the bulk GMO (*a*_o_ = 5.318 Å, *b*_o_ = 7.431 Å and *c*_o_ = 5.866 Å)^21,24^. Moreover, the cell parameters of the tetragonal film phase undergo a remarkable alteration analog to an expansion of ~2.8% in *b* and ~6.4% in *a*, while *c* contracts ~3.2%. Such significant values should be considered beyond a mere distortion of the orthorhombic phase.

**Figure 3:**
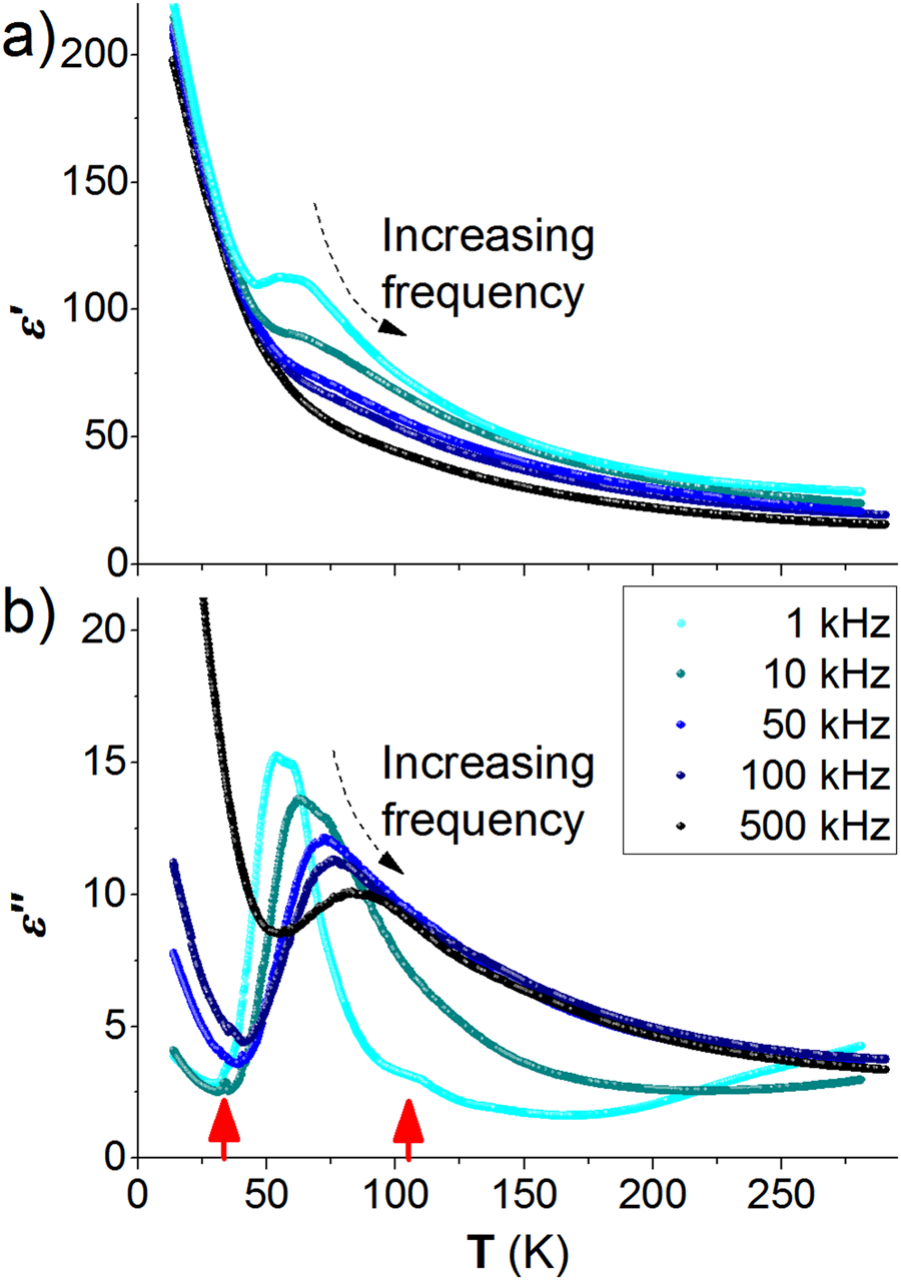
Temperature dependence of the (**a**) real and (**b**) imaginary parts of the complex electric permittivity, measured at different fixed frequencies. Red arrows point to anomalies of *ɛ*”(T).

Figure 3(a,b) shows the temperature dependence of the real (*ɛ*’) and imaginary (*ɛ*”) parts of the complex electric permittivity of the GMO thin film, measured at different fixed frequencies.

The real part of the electric permittivity exhibits dispersion for temperatures above 30 K, having a higher magnitude in the 30–150 K temperature range. The shapes of the *ɛ*’(T) curves are frequency dependent, where the maximum anomalous behavior is obtained for the *ε*’(T) curve measured at 1 kHz. As the frequency increases, the amplitude of the anomaly decreases and moves towards higher temperatures. In addition, the observed anomalies in the imaginary part of *ɛ*”(T) are also frequency dependent in the whole studied temperature range. These features in *ε*’(T) and *ε*”(T) evidence for the existence of a dielectric relaxation process in GMO thin film, whose activation energy is 80 meV, higher than the reported value 22 meV found in bulk GMO^25^. The *ɛ*”(T) curve measured at 1 kHz exhibits a small anomaly at 105 K, which is associated with the antiferrodistortive phase transition of the STO substrate. Due to the relatively small amplitude of this anomaly and the fact that it is only observed at a single frequency along with the different temperature dependence of the measured dielectric constant regarding the actual dielectric response of STO, we realize that the contribution of the STO to the overall dielectric response is negligible small. A closer look at the *ɛ*”(T) curve measured at 10 kHz reveals a small but clear anomaly at T_1_ near 35 K. The results here described strongly contrast with the ones reported for orthorhombic GMO thin films onto (010)-YAlO_3_, where a cusp-like anomaly in *ɛ*’(T) curve is observed^15^. Though the temperatures corresponding to the anomalies in *ɛ*’(T) and *ɛ*”(T) curves found in this work and in ref. ^15^ are similar, the difference between the *ɛ*’(T) curve shapes evidences for different mechanisms underlying the dielectric properties of tetragonal and orthorhombic GMO films^15,16^.

**Figure 4:**
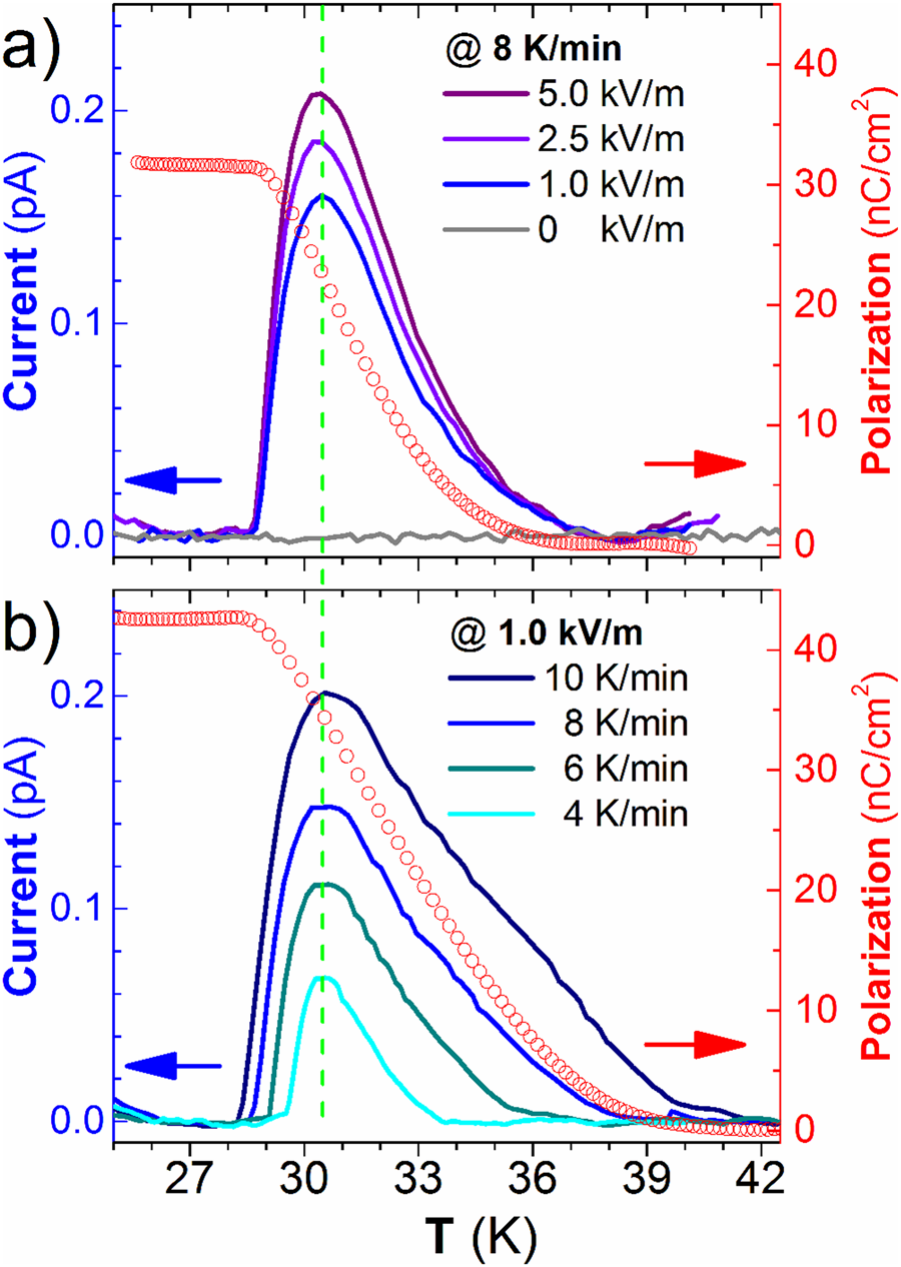
Left axis: TSDC as a function of temperature, after cooling the sample with different (**a**) polling electric fields and (**b**) heating rates. Right axis: the respective estimated polarization, obtained from time integration of the highest TSDC density.

In order to disentangle the underlying processes that can be associated with the emergence of the anomaly observed at T_1_, we have studied the polar properties of the tetragonal GMO thin film. For this purpose, we have measured the thermally stimulated depolarizing current (TSDC) in heating runs, at fixed temperature rate of 8 K/min, in two distinct poling conditions: the sample was cooled i) in the absence of an electric field, and ii) under different bias electric fields: 1.0; 2.5; and 5.0 kV/m. The TSDC was measured in the absence of an applied electric field. The results are displayed in Fig. 4(a).

In the absence of a polling electric field, no anomaly in the temperature dependence of the TSDC could be observed. An anomaly in the temperature dependence of the TSDC is apparent at 32 K, in experiments for which the sample was cooled down under the poling field, while experiments using inverse poling fields result in the respective reversed TSDC signal. Moreover, the temperature for which the anomaly in the temperature dependence of the TSDC is ascertained does not depend on the strength of the poling electric field. However, as it is expected, the amplitude of the anomaly increases by increasing the poling electric field. The heating rate dependence of TSDC was also studied after poling the film with a 1.0 kV/m electric field, in order to determine its actual origin. As it can be seen in Fig. 4(b), the amplitude of TSDC increases and becomes broader with increasing heating rate, possibly due to thermal inertia effects of the measuring system, but the peaks maxima, signaled by the vertical green dotted line, remain at 32 K, independently of the heating rate. This outcome reflects the spontaneous origin of the TSDC associated with the stabilization of a ferroelectric phase, and excludes others charge displacement origins, as those observed in electrets and related compounds, wherein the driving force is the restoration of charge neutrality through disorientation of electric dipoles, and diffusion of immobilized space charges, among others^26^. In order to determine the temperature dependence of the in-plane component of the electric polarization associated with the ferroelectric phase, we have carried out the time integration of the pyroelectric current from Fig. 4(b) that was measured at the lowest electric poling field, in order to avoid induced contributions expected from applying higher poling fields. In this case, the in-plane component of the electric polarization value around 40 nC.cm^−2^ is obtained below 32 K, of similar order of the saturation values found in bulk TbMnO_3_ and DyMnO_3_ below 20 K^9^. Shimamoto *et al*. have reported the emergence of an electric polarization of ∼1μC.cm^−2^ along the *a*-axis in 10 nm thick orthorhombic GMO thin films onto (010)-YAlO_3_ substrates^15^. This value is more than one order of magnitude larger than the one found in the tetragonal GMO films, which can be explained by the different nature of the ferroelectric phase in orthorhombic and tetragonal GMO films, ascertained by the different temperature dependences of the electric permittivity. Moreover, the difference between the values referred to above also stems from the fact that we were only measuring the in-plane component of the electric polarization.

Magnetic measurements performed by SQUID in the thin film samples are not accurate enough to conclude about the magnetic properties of the tetragonal GMO film (for details, see *Supplemental Material*). The magnetic contribution of the GMO phase, subtracted from the diamagnetic signal of the substrate, shows no hysteresis and a small paramagnetic signal (χ ~3.10^−3^ at 5 K). Besides, no relevant anomalies are observable near 32 K.

Previously published works on several rare-earth manganites thin films (GdMnO_3_, TbMnO_3_, HoMnO_3_) generally report that a distorted orthorhombic structure is obtained using STO(001) substrates^17,27–30^. Contrarily, we have obtained in a totally strained epitaxial GMO film, a previously unprecedented tetragonal structure up to a 35 nm thickness. It is worth to note that such a tetragonal like structure was also obtained in a TbMnO_3_ film, though up to a much less thickness of 2 nm^28^. The observation of a pyroelectric current in thicker orthorhombic GMO films onto STO(001), whose temperature dependence resembles the one observed in this work^17^, points out for a common source of the improper ferroelectric phase in GMO films with different structures. Though tempting, it is not at all adequate to visualize the behavior of such GMO tetragonal films using the same interpretative models found for the orthorhombic rare-earth manganites^31,32^. Actually, the tetragonal symmetry of GMO thin film imposes constrains to the packing and mobility of the MnO_6_ octahedra, corresponding to significant rearrangements on the Mn-O-Mn orbitals overlap and respective alterations in charge transfer and magnetic interactions. Moreover, the contribution of twin-like domain structure ascribed to the high temperature electric polarization in orthorhombic GMO films^17^ is not apparently applicable in our case, since such domain structure is not observed in tetragonal GMO films. Thus, the proposed mechanisms to explain the stabilization of the ferroelectric phase in orthorhombic GMO thin films have to be revised.

Summarizing, an earlier not reported tetragonal phase in epitaxially strained GdMnO_3_ films onto STO(001), with a thickness up to 35 nm, was ascertained. Another remarkable outcome is the stabilization of a ferroelectric phase below 32 K. From the relative low saturation value of the in-plane component of the spontaneous electric polarization and the broad shape of the corresponding pyroelectric anomaly, we are led to conclude that the ferroelectric phase has an improper character. The similar temperature dependent TSDC curves obtained in orthorhombic GMO onto STO(001) suggest a common origin of the ferroelectric phase, whose mechanisms deserve further studies. Thus, this work provides a guide, through strain engineering, to unravel new and unexpected phases in perovskite thin films.

## Methods

High quality GdMnO_3_ polycrystalline targets were prepared using urea sol-gel combustion method^33^. The phase formation, chemical composition, crystallographic structure and microstructure were fully characterized. Details of target preparation and characterization can be found elsewhere^33^. Polished STO(001) substrates were previously cleaned before the deposition, in ultrasonic baths to remove dust particles and greases using acetone, isopropanol and bi-deionized water, for 15 min each, respectively. The substrates were then dried using nitrogen flow. GMO thin films were deposited onto STO(001), through RF-magnetron sputtering, working at 13.56 MHz and 80 W RF power. The distance between target and substrate was fixed at 130 mm. The substrate was kept at 800 °C during deposition, and temperature was measured with a *K*-type thermocouple positioned directly behind the substrate. Prior to deposition, the chamber was evacuated to 5 × 10^−6^ mbar by a turbomolecular pump. The deposition rate of GMO was estimated to be around 2 nm/min. During film deposition, the pressure inside the chamber was adjusted to 5 × 10^−2^ mbar, with a gas composition setting a ratio of Ar:O_2_ of 4:1. After deposition, the films were left to cool naturally to room temperature in a pure oxygen atmosphere of 1 mbar, without any annealing step.

The phase formation and crystallographic structure of the GMO thin films were analyzed by high resolution X-ray diffraction (HR-XRD) at room conditions, using a *X’Pert MRD Philips* four-circle diffractometer, based in a Bragg–Brentano para-focusing optics configuration, operating with Cu Kα radiation ***λ*** = 1.54056 Å at 30 mA and 40 kV, for performing conventional theta-2theta (Θ-2Θ***)*** scans, pole figures and reciprocal space maps (rsm). Analysis of the diffractograms was performed through the basic Le Bail mode for assessment of crystallographic structure and parameters of the film. The surface morphology, cross-section and chemical composition of the thin film samples was checked using scanning electron microscopy (SEM) in a *Quanta 400 FEG ESEM/EDAX Genesis X4M*, using 15.00 kV for secondary electrons mode and for backscattering mode. X-ray photoelectron spectroscopy (XPS) was performed using a *Kratos Axis Ultra HSA* equipment with 15.00 kV source power, in order to analyze the surface composition and electronic state of elements. Atomic force microscopy was performed in contact mode using a *NT-MDT Ntegra Aura* and respective *Nova_Px 3.4.0* interface software. Commercial Pt coated doped silicon probes from *NT-MDT*, with curvature radius of 10 nm, resonance frequency ~130 kHz and spring constant of 3 N/m, were used. The topographic images were edited via *WSxM 5.0 8.0* software. The temperature dependence of the complex electric permittivity was measured in the 1 kHz - 1 MHz frequency range with an *HP4284A* impedance analyzer, under an *ac* electric field of amplitude 50 V/cm, in the 10–300 K temperature range. The thermally stimulated depolarization current (TSDC) was measured as a function of temperature, with a standard short-circuit method, using a *Keithley 617* electrometer, with a resolution of 0.1 pA. The current intensity was measured during heating run in short-circuit conditions, for different heating rates. Different poling fields were applied during the preceding cooling process and a 30 min short-circuit was performed at the lowest temperature and before current measurements, in order to release spurious charges at the electrodes. The temperature dependence of the electric polarization was obtained by time integration of the TSDC current. The electric polarization was measured in the in-plane film orientation. As the electrodes are parallel to the substrate edges, which in turn are parallel to the axes of the STO cubic cell, and the GMO film has the in-plane crystallographic axes at 45° relatively to the STO axes, the electric polarization was measured along the [110] tetragonal direction. Both electric permittivity and TSDC were measured using interdigital electrodes (IDEs), fabricated on the prepared films by conventional photolithography process to define the resist pattern. The *AZ 1505 Microchemicals* photoresist was first spread over the film with a spinner *Headway Research Inc PWM32-PS-R790* and then dried by soft bake in a hotplate at 100 °C during 1 minute. After exposure using a *Heidelberg Direct Laser Writer uPG10* and development with an appropriate solvent. The required IDEs pattern was used as a mask to remove the surplus material by ion milling in a *Nordiko3600* system with a broad Argon beam of 40 mA (~130 μA/cm^2^) at a pressure of 0.2 mTorr.

The IDEs (see Fig. S1 of Supplementary Material) consist of N = 50 gold electrodes, deposited by evaporation using an *Edwards e-beam evaporators Auto 306*, filling the trenches previously made by ion beam etching and after removed by lift-off the resist and the corresponding excess of gold. These electrodes have length L = 7 mm, width W = 20 μm, interspacing G = 20 μm and depth of about 90% of the thickness of the deposited film, in order to avoid a significant dielectric response coming from the substrate. The lithographed IDEs on the prepared GMO films (see Fig. S2 of Supplementary Material) shows a regular pattern of 20.0 ± 0.5 µm wide channels and steps. This technique is fundamental to optimize electric measurements in the film phase across multiple and broader 20 μm region between IDEs, rather than to be limited to gauge the film thickness (<<100 nm) between top and bottom electrodes. Moreover, rare-earth manganites are known to be relatively prone to current losses or even tunneling within such nanometer scale, which would hamper dielectric and polar measurements. As the electrodes are parallel to the substrate edges, which in turn are parallel to the axes of the STO cubic cell, and the GMO film has the in-plane crystallographic axes at 45° relatively to the STO axes, the electric polarization was measured along the [110] direction.

## Supplementary information

Supplementary information
