# Supplementary material for: Strain-Engineered Tetragonal Phase and Ferroelectricity in GdMnO3 Thin Films Grown on SrTiO3 (001)
Source: Sci Rep. 2019 Dec 10;9:18755. doi: 10.1038/s41598-019-55227-2 (PMC6904445; doi:10.1038/s41598-019-55227-2)
Supplement: Supplementary file 1 — Supplementary information [file 41598_2019_55227_MOESM1_ESM.docx]

**Supplemental Material**

Strain-Engineered Tetragonal Phase and Ferroelectricity in GdMnO_3_ Thin Films Grown on SrTiO_3_ (001)

P. Machado ^1^, F. G. Figueiras ^1^, R. Vilarinho ^1^, J. R. A. Fernandes ^2^, P. B. Tavares ^3^, M. Rosário Soares ^4^, S. Cardoso ^5^, J. P. B. Silva^6^, A. Almeida ^1^, J. Agostinho Moreira ^1^

^1^ IFIMUP and Departamento de Física e Astronomia, Faculdade de Ciências, Universidade do Porto. R. Campo Alegre, 687, 4169-007 Porto. Portugal.

^2^ CQVR & Physics Department, University of Trás-os-Montes e Alto-Douro, Ap.º 1013, 5001-801 Vila Real, Portugal

^3^ CQVR & Chemistry Department, University of Trás-os-Montes e Alto-Douro, Ap.º 1013, 5001-801 Vila Real, Portugal

^4^ CICECO & LCA, University of Aveiro, 3810-193 Aveiro, Portugal.

^5^ INESC-MN e Instituto Superior Técnico, Universidade de Lisboa, Rua Alves Redol 9, 1000-029 Lisboa, Portugal.

^6^ Centro de Física das Universidades do Minho e do Porto (CF-UM-UP), Campus de Gualtar, 4710-057 Braga, Portugal

Corresponding author: jamoreir@ fc.up.pt

**Morphology and chemical composition**

Figure S1 shows a representative cross-section SEM image of the as-grown thin film GMO/STO(001) deposited during 20 minutes. The film exhibits a dense and uniform layer, with a good adhesion to the substrate, without structural defects or cracks and average thickness of 35 ± 1 nm. EDS measurements confirm the presence of the elements Gd and Mn in ratios between 0.75 and 1.15, conditional to the incident electrons accelerating voltage (15-30 kV). Hence Gd:Mn ratio can be estimated close to 1, within a 20% error margin for both Gd and Mn atomic composition. This imprecision in composition is expected due to the hindering diffraction effects of the film reduced thickness and interface with substrate [1].

The XPS spectrum of the binding energy of the Mn 3*s* orbitals enables to distinguish the Mn oxidation states [2]. As seen in Figure S2, the characteristic splitting between the correlated local maxima of the multiplet peaks are estimated between 5.0 and 5.3 eV, corresponding to the overall presence of Mn^3+^, and even of higher ionicity forms, associated with shorter Mn-O distances found in films, as described by Rubi *et al*. [3]. The XPS spectrum shows no traces of the reduced Mn^2+^, which would be formed if oxygen vacancies were present.

Figure S3(a) shows a demonstrative AFM topography image and respective histogram of the as-grown ~35 nm thin film. These scans allowed inspecting both local and overall film topography, confirming the smooth and homogeneous quality of the film surface. The film average roughness within a representative 2 x 2 µm^2^ area was estimated to be near 0.3 nm, which is within the scale of the STO lattice cell. The quality and precision of the subsequent lithography process is also exemplified by the AFM results shown in figure S3(b). In the 150 x 50 µm^2^ (512 x 168 px.) scan is possible to observe well-defined terraces and grooves along with representative normalized profiles evidencing the regular and sharp pattern of steps and channels of 20.0 ±0.3 µm wide and of 25 ±1 nm deep. Due to the etching procedure on the GMO film, the bottom surface of the grooves exhibits an average roughness near 2 nm and the carved walls have a slight 3º inclination inward. The later result confirms that these groves do not reach the substrate, ensuring that the subsequent Au electrodes will not be in direct contact with STO film in order to prevent contributions of the substrate to the dielectric response of the film. The schematics and the SEM image of the lithographed IDEs prepared GMO films are shown in Figures S4 and S5.

| 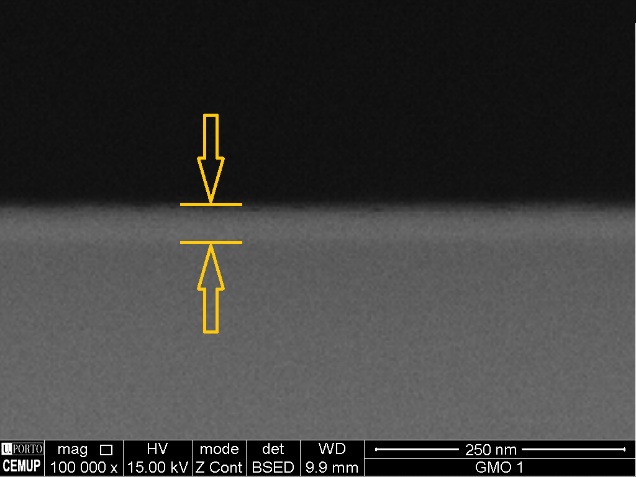  **Figure S1.** Representative SEM cross-section image of the as-processed GMO thin film onto STO(001). |   **Figure S2:** XPS spectrum in the 75 – 100 eV energy range. |
| --- | --- |
| 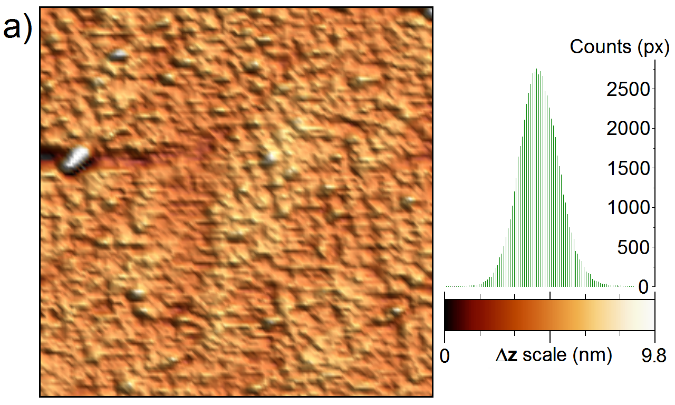 | 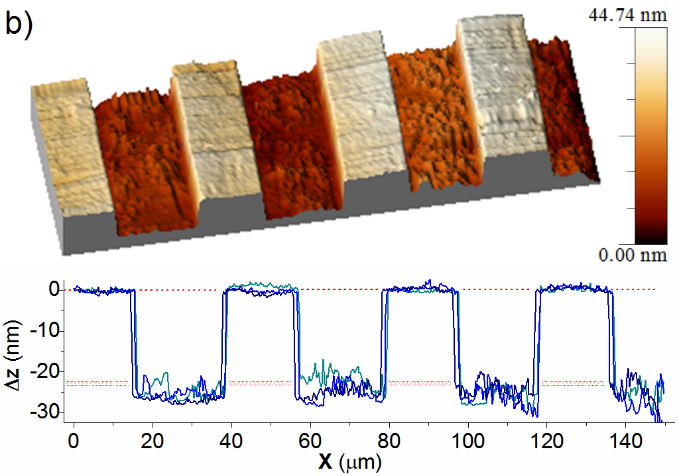 |
| **Figure. S3:** AFM topographic scan (a) over 2 x 2 µm^2^ area with respective histogram and  (b) over 150 x 50 µm^2^ area of the lithographed film with representative line histograms [4]. | |
| 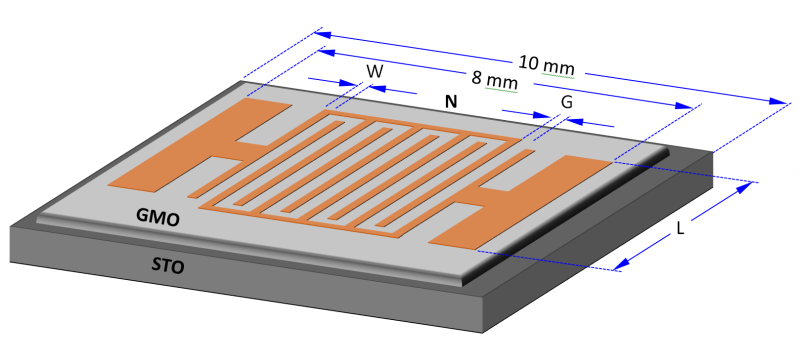  **Figure S4.** Schematic representation of the IDEs  (N = 50, G = W = 20 μm, L = 7 mm). | 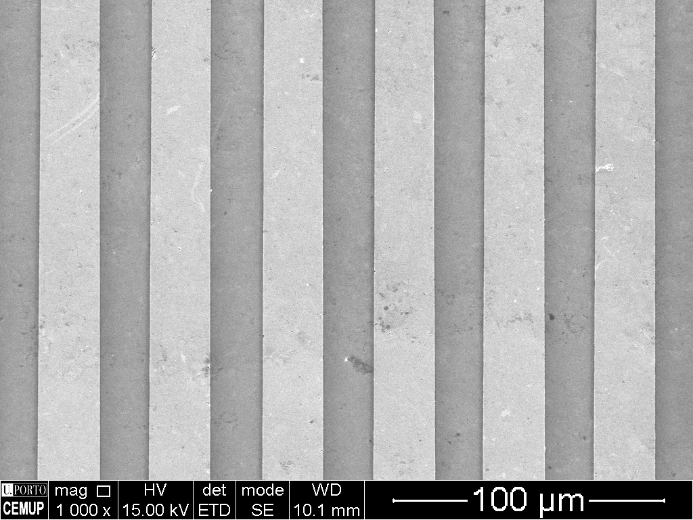  **Figure S5.** Representative SEM image of the surface of the GMO thin films after the etching by Ion Beam. |

**Magnetic measurements**

Magnetic measurements performed by SQUID in the thin film samples are not accurate enough to conclude about the magnetic properties of the tetragonal GMO film. The magnetic response of the STO(001) substrate and GMO/STO(001) thin films was measured in field cooling (FC) conditions, and the results are shown in Fig. S6. In these measurements, the external field was applied parallel to the plane film and substrate. Regarding the results obtained in STO, the M(T) values are within the experimental sensibility of the SQUID machine ( 1x10^-7^ emu). However, small variations could be ascertained in this measurement. The data concerning the 35 nm thick GMO/STO(001) film exhibit a small kink at 53K and M(T) monotonous increase as temperature decreases. It is worth to stress that the kink observed occurs at the same temperature for which M(T) curve of STO exhibits a change of slope. Thus, we consider that the quality of the magnetization measurement results hinders us to make claims about the magnetism of the films. However, as can be seen from curve M (T) shown in Fig. S6, the increase of M(T) as temperature decreases points out for the ordering of the Gd^3+^ spins.

**Figure S6:** Temperature dependence of the magnetization of the GMO thin film deposited onto STO(001) (black points) and clean STO substrate (red points), measured in field cooling conditions.

**References**

[1] T. Yamanaka, N. Hirai, Y. Komatsu. Structure change of Ca_1-x_Sr_x_TiO_3_ perovskite with composition and pressure. American Mineralogist 87, 1183 (2002).

[2] M.C. Besinger, Brad P. Payne, A. P. Grosvenor, Leo W.M. Lau, A. R. Gerson, Roger St.C. Smart. Resolving surface chemical states in XPS analysis of first row transition metals, oxides and hydroxides: Cr, Mn, Fe, Co and Ni. App. Sur. Sci. 257, 2717 (2011).

[3] D. Rubi, C. de Graaf, C.J.M. Daumont, D. Mannix, R. Broer, B. Noheda. Ferromagnetism and increased ionicity in epitaxially grown TbMnO_3_ films. Phys. Rev. B 79, 014416 (2009).

[4] I. Horcas, R. Fernandez, J.M. Gomez-Rodriguez, J. Colchero, J. Gomez-Herrero, and A.M. Baro. WSXM: A Software for Scanning Probe Microscopy and a Tool for Nanotechnology. Review of Scientific Instruments 78, 013705 (2007).
